# Genotypes of *Cryptosporidium* spp., *Enterocytozoon bieneusi* and *Giardia duodenalis* in dogs and cats in Shanghai, China

**DOI:** 10.1186/s13071-016-1409-5

**Published:** 2016-03-01

**Authors:** Hailing Xu, Yue Jin, Wenxian Wu, Pei Li, Lin Wang, Na Li, Yaoyu Feng, Lihua Xiao

**Affiliations:** State Key Laboratory of Bioreactor Engineering, School of Resources and Environmental Engineering, East China University of Science and Technology, Shanghai, 200237 China; Division of Foodborne, Waterborne, and Environmental Diseases, National Center for Emerging and Zoonotic Infectious Diseases, Centers for Disease Control and Prevention, Atlanta, GA 30333 USA

**Keywords:** *Cryptosporidium* spp., *Enterocytozoon bieneusi*, *Giardia duodenalis*, Genotype

## Abstract

**Background:**

Controversies exist on the potential role of companion animals in the transmission of enteric pathogens in humans. This study was conducted to examine the genotype distribution of *Cryptosporidium* spp., *Enterocytozoon bieneusi*, and *Giardia duodenalis* in companion animals in Shanghai, China, and to assess their zoonotic potential.

**Methods:**

Fecal specimens from 485 dogs and 160 cats were examined for the occurrence and genotype distribution of the three pathogens by PCR. PCR products were sequenced to determine the species and genotypes. The χ^2^ test was used to compare differences in infection rates between living conditions or age groups.

**Results:**

*Cryptosporidium* spp., *E. bieneusi* and *G. duodenalis* were found in 39 (8.0 %), 29 (6.0 %) and 127 (26.2 %) of dogs, and 6 (3.8 %), 9 (5.6 %) and 21 (13.1 %) of cats, respectively. Infection rates of the pathogens in dogs from pet shops and a clinic were higher than those in household dogs, and higher in cats from one animal shelter than from pet shops. No significant differences in infection rates were detected among age groups. *Cryptosporidium canis* and *C. felis* were the only *Cryptosporidium* species found in dogs and cats, respectively. *Enterocytozoon bieneusi* genotype PtEb IX was the dominant genotype in dogs, whereas Type IV and D were the most common ones in cats. Multi-locus sequence typing at the glutamate dehydrogenase, β-giardin, and triosephosphate isomerase loci revealed the presence of *G. duodenalis* assemblages A (*n* = 23), B (*n* = 1), C (*n* = 26), and D (*n* = 58) in dogs (only A in household dogs) and assemblages A (*n* = 2), B (*n* = 6), C (*n* = 2), D (*n* = 1), and F (*n* = 7) in cats. Co-infection was detected in 24 dogs and 5 cats, especially those living in crowded conditions.

**Conclusions:**

Living condition is a major risk factor affecting the occurrence of enteric protists in companion animals in China, and although dogs and cats can be potential sources of human infections, the different distribution of pathogen species and genotypes between dogs and cats suggests that inter-species transmission of these pathogens is probably rare in the study area.

## Background

*Cryptosporidium* spp., *Enterocytozoon bieneusi* and *Giardia duodenalis* are protist pathogens in humans and diverse farm, companion, and wild animals, causing acute or chronic diarrhea and other gastrointestinal symptoms. Humans obtain these pathogens after exposures to infected persons (anthroponotic transmission) or animals (zoonotic transmission) or ingestion of contaminated food or water (food-borne or water-borne transmission) [1–3]. Among the near 30 established *Cryptosporidium* species, *C. hominis*, *C. parvum*, *C. meleagridis*, *C. canis*, and *C. felis* are responsible for most human cryptosporidiosis cases, with the latter two being zoonotic species in dogs and cats, respectively. *Enterocytozoon bieneusi* has at least eight genotype groups, with Group 1 containing the common zoonotic genotypes and Groups 2 - 8 containing mostly host-adapted genotypes [4–6]. Similarly, *G. duodenalis* is consisted of the zoonotic assemblages A and B and host-adapted assemblages C to H [2].

Recent genotyping investigations of *Cryptosporidium* spp., *E. bieneusi* and *G. duodenalis* in companion animals such as dogs and cats have identified the occurrence of numerous zoonotic species/genotypes/assemblages in these animals, such as *C. canis*, *C. felis*, *E. bieneusi* genotypes D, Type IV, Peru10, and WL11 and *G. duodenalis* assemblages A and B [1, 2, 7]. Few of these studies, however, involved sampling of humans at the same locations. In China, there are only a few molecular epidemiological surveys of these pathogens in dogs or cats in Henan, Guangdong, Heilongjiang, Liaoning, Sichuan, and Shaanxi provinces [8–14], whereas comparable data from humans are mostly not available. Most of these studies involved characterization of one pathogen in one species of animal.

In recent molecular epidemiological studies of these three pathogens in in-hospital children and waste-water in Shanghai, China, we identified common occurrence of some zoonotic species or genotypes, such as *C. canis*, *C. felis*, *E. bieneusi* genotype D and Type IV, and *G. duodenalis* assemblages A and B, which might be harbored by dogs and cats [15–17]. Shanghai is the largest city with the highest population density in mainland China, and has over three million pet dogs and cats. With the rapid development of the pet industry, increasing numbers of pet shops and veterinary clinics are established in the city, some of which have less than desirable hygiene conditions. In the present study, we examined the occurrence of *Cryptosporidium* spp., *E. bieneusi* and *G. duodenalis* in pet dogs and cats in different living conditions in Shanghai, and assessed the zoonotic potential of these pathogens at the genotype level.

## Methods

### Specimens

During 2011 to 2014, 645 fecal specimens were obtained from dogs and cats in urban areas of Shanghai, China, including the Xuhui and Minhang Districts in the southwestern city and Putuo District in the northwestern city. Altogether, 485 fecal specimens were collected from dogs, including 102 specimens from household dogs, 61 specimens from dogs in a veterinary clinic, and 322 specimens from dogs in pet shops. By living condition, household dogs generally lived in a cleaner environment than dogs in the veterinary clinic and pet shops. These dogs were divided into two age groups: < 6 months (*n* = 121) and 6–24 months (*n* = 20), with the remaining of unknown age (*n* = 344). Likewise, 160 fecal specimens were collected from cats in pet shops (*n* = 120) and an animal shelter (*n* = 40), being divided into two age groups: < 6 months (*n* = 66) and 6–24 months (*n* = 83), with a few of unknown age (*n* = 11). The sanitary condition of pet shops was better than the one in the animal shelter. One specimen per animal was used for this study. Fecal specimens were collected using plastic bags, transferred into 50 ml centrifuge tubes and preserved in 2.5 % potassium dichromate at 4 °C.

### DNA extraction and PCR

After washing 200 μl of fecal specimens twice with distilled water by centrifugation, DNA was extracted from them using the Fast DNA SPIN Kit for Soil (MP Biomedical, CA) following manufacturer-recommended procedures. The extracted DNA was stored at −20 °C until being analyzed by PCR. For the detection of *Cryptosporidium* spp. and *E. bieneusi*, a 587-bp fragment of the small-subunit (SSU) rRNA gene [18] and a 392-bp fragment covering the entire internal transcribed spacer (ITS) region of the rRNA gene [19] were amplified by nested PCR, respectively. *Giardia duodenalis* was detected by nested PCR targeting the glutamate dehydrogenase (*gdh*) [20], β-giardin (*bg*) [21], and triosephosphate isomerase (*tpi*) [22] genes. Two replicates were used in PCR analysis of each specimen at each locus. The secondary PCR products were examined by electrophoresis in 1.5 % agarose gels and visualized after ethidium bromide staining.

### Sequence analysis

All positive secondary PCR products were sequenced in both directions on an ABI 3730 (Applied Biosystems, Foster City, CA) at the BioSune Biotechnology Company (Shanghai, China). To determine the pathogen species or genotypes, sequences obtained were assembled using ChromasPro 1.5 (http://www.technelysium.com.au/ChromasPro.html), edited using BioEdit 7.1 (http://www.mbio.ncsu.edu/BioEdit/bioedit.html), and aligned with each other and reference sequences of each genetic target downloaded from GenBank using Clustal X 1.81 (http://www.clustal.org/). Neighbor-joining trees of nucleotide sequences from *G. duodenalis* were constructed using genetic distances from the Kimura-2 parameter model and the software Mega 6.06 (http://www.megasoftware.net/). Representative nucleotide sequences generated in this study were deposited in GenBank under accession numbers KU156631 - KU156669.

### Data analysis

The χ^2^ test implemented in SPSS version 17.0 (SPSS Inc., Chicago, IL, USA) was used to compare differences in infection rates between living conditions or age groups. Differences with *P* < 0.05 were considered significant.

## Results

### Occurrence of *Cryptosporidium* spp., *E. bieneusi*, and *G. duodenalis*

*Cryptosporidium* spp., *E. bieneusi* and *G. duodenalis* were detected in 39 (8.0 %), 29 (6.0 %), and 127 (26.2 %) of the 485 canine specimens, and 6 (3.8 %), 9 (5.6 %), and 21 (13.1 %) of the 160 feline specimens, respectively. There was no obvious difference in fecal consistency between positive and negative specimens for any of the pathogens. Infection rates of *Cryptosporidium* spp., *E. bieneusi* and *G. duodenalis* in dogs and cats from different age groups and living conditions are shown in Table 1. When all three pathogens were considered together, living condition-associated differences were found in both dogs and cats. Overall infection rates of three pathogens in dogs in the veterinary clinic (59.0 %) and pet shops (37.6 %) were significantly higher than the infection rate in household dogs (12.7 %; *P* < 0.05). Similarly, the infection rate in cats from the animal shelter (30.0 %) was significantly higher than the one in cats from pet shops (15.8 %; *P* < 0.05).

**Table 1:** Occurrence of *Cryptosporidium* spp., *Enterocytozoon bieneusi*, and *Giardia duodenalis* in dogs and cats by age and sample source

Host	Age/Source	No. of specimens	No. of positives (positive rate; 95 % confidence interval)		
			*Cryptosporidium* spp.	*E. bieneusi*	*G. duodenalis*
Dogs	<6 months	121	13 (10.7 %; 0.049–0.166)	5 (4.1 %; 0.005–0.078)	45 (37.2 %; 0.263–0.481)
	>6 months	20	0 (0 %)	3 (15.0 %; −0.020–0.320)	5 (25.0 %; 0.031–0.469)
	Unknown	344	26 (7.6 %; 0.047–0.105)	21 (6.1 %; 0.035–0.087)	77 (22.4 %; 0.174–0.274)
	Veterinary clinic	61	8 (13.1 %; 0.040–0.222)	2 (3.3 %; −0.013–0.078)	33 (54.1 %; 0.356–0.726)
	Households	102	0 (0 %)	8 (7.8 %; 0.024–0.133)	7 (6.9 %; 0.018–0.119)
	Pet shops	322	31 (9.6 %; 0.062–0.130)	19 (5.9 %; 0.032–0.086)	87 (27.0 %; 0.213–0.327)
	Subtotal	485	39 (8.0 %; 0.055–0.106)	29 (6.0 %; 0.038–0.082)	127 (26.2 %; 0.216–0.307)
Cats	<6 month	66	3 (4.5 %; −0.006–0.097)	4 (6.1 %; 0.001–0.120)	9 (13.6 %; 0.047–0.225)
	>6 month	83	3 (3.6 %; −0.005–0.077)	5 (6.0 %; 0.007–0.113)	10 (12.0 %; 0.046–0.195)
	Unknown	11	0 (0 %)	0 (0 %)	2 (18.2 %; −0.070–0.434)
	Shelter	40	3 (7.5 %; −0.010–0.160)	4 (10.0 %; 0.002–0.198)	7 (17.5 %; 0.045–0.305)
	Pet shops	120	3 (2.5 %; −0.003–0.053)	4 (3.3 %; 0.001–0.066)	14 (11.7 %; 0.056–0.178)
	Subtotal	160	6 (3.8 %; 0.007–0.068)	9 (5.6 %; 0.020–0.093)	21 (13.1 %; 0.075–0.187)
Total		645	45 (7.0 %; 0.049–0.090)	38 (5.9 %; 0.040–0.078)	149 (23.1 %; 0.194–0.268)

For *Cryptosporidium* spp., dogs in the clinic (13.1 %) and pet shops (9.6 %) had significantly higher infection rates than household dogs (0.0 %; *P* < 0.01). Similarly, dogs in the clinic (54.1 %) and pet shops (27.0 %) also had significantly higher *G. duodenalis* infection rates than household dogs (6.9 %; *P* < 0.01). However, there was no significant living condition-associated difference in dogs in *E. bieneusi* infection rates. For *G. duodenalis*, different positive rates were observed among the *gdh*, *bg*, and *tpi* loci. The positive rates were 15.3 %, 17.7 %, and 8.7 % in dogs and 9.4 %, 7.5 %, and 7.5 % in cats, respectively.

There was no significant difference in overall or pathogen-specific infection rates between the two age groups of both dogs and cats (*P* > 0.05).

### *Cryptosporidium* species in dogs and cats

*Cryptosporidium* species detected in dogs and cats are shown in Table 2. DNA sequencing was successful for all 39 PCR-positive canine specimens, and identified the presence of only *C. canis*. In feline specimens, four of six positive PCR products were successfully sequenced and identified as *C. felis*. The partial sequences from the remaining two also belonged to *C. felis*. Nucleotide sequences of nine *C. canis* specimens and one *C. felis* specimen were identical to GenBank reference sequences KF516543 and KJ194110, respectively. Nucleotide sequences obtained from the remaining 30 *C. canis* specimens had minor differences from the reference sequence KF516543, including one single nucleotide polymorphism (SNP) in 24 specimens (T to C substitution at position 627) and 2 SNPs in six specimens (A to G substitution at position 473 and T to C substitution at position 627). Likewise, the remaining three *C. felis* sequences had minor differences from KJ194110, including 1 SNP (T to A substitution at position 456) in one specimen, 1 SNP (T to A substitution at position 456) and one T deletion at position 486 in one specimen, and 1 SNP (T to A substitution at position 456), three T deletions at position 445–447, and one T deletion at position 486 in one specimen.

**Table 2:** Species/genotypes/assemblages of *Cryptosporidium* spp., *Enterocytozoon bieneusi*, and *Giardia duodenalis* in dogs and cats by age and sample source

Host	Age/Source	No. of specimens	Species/genotypes/assemblages (no. of specimens)		
			*Cryptosporidium* spp.	*E. bieneusi*	*G. duodenalis*
Dogs	<6 months	121	*C. canis* (13)	PtEb IX (4), D (1)	A (1), C (8), D (29), D/C (4), A/D (1)
	>6 months	20		PtEb IX (3)	C (3), D (2)
	Unknown	344	*C. canis* (26)	PtEb IX (21)	A (22), B (1), C (15), D (27), C/D (6), A/C (2)
	Veterinary clinic	61	*C. canis* (8)	PtEb IX (2)	A (2), C (3), D (18), C/D (6)
	Households	102		PtEb IX (8)	A (7)
	Pet shops	322	*C. canis* (31)	PtEb IX (18), D (1)	A (14), B (1), C (23), D (40), C/D (4), A/C (2), A/D (1)
	Subtotal	485	*C. canis* (39)	PtEb IX (28), D (1)	A (23), B (1), C (26), D (58), C/D (10), A/C (2), A/D (1)
Cats	<6 months	66	*C. felis* (3)	Type IV (2), D (2)	A (1), B (2), C (2), D (1), F (3)
	>6 months	83	*C. felis* (3)	Type IV (3), D (2)	A (1), B (4), F (3)
	Unknown	11			F (1)
	Shelter	40	*C. felis* (3)	Type IV (2), D (2)	A (1), B (4), F (2)
	Pet shops	120	*C. felis* (3)	Type IV (3), D (2)	A (1), B (2), C (2), D (1), F (5)
	Subtotal	160	*C. felis* (6)	Type IV (5), D (4)	A (2), B (6), C (2), D (1), F (7)

### *E. bieneusi* genotypes in dogs and cats

Sequence analysis of the ITS products revealed the presence of *E. bieneusi* genotypes PtEb IX (*n* = 28) and D (*n* = 1) in dogs, and Type IV (*n* = 5) and D (*n* = 4) in cats (Table 2). Nucleotide sequences of PtEb IX, Type IV and D detected in this study were identical to those deposited in GenBank under accession numbers DQ885585, KF305582, and KF305583, respectively.

### Assemblages and subtypes of *G. duodenalis* in dogs and cats

DNA sequencing was successful for 121 of 127 *Giardia*-positive canine specimens and 18 of 21 *Giardia*-positive feline specimens. Multi-locus sequence typing at *gdh*, *bg*, and *tpi* loci revealed the presence of *G. duodenalis* assemblages A (*n* = 23), B (*n* = 1), C (*n* = 26), and D (*n* = 58) in dogs, and assemblages A (*n* = 2), B (*n* = 6), C (*n* = 2), D (*n* = 1), and F (*n* = 7) in cats (Table 2). Concurrent infections of mixed assemblages were detected in 13 dogs, including A/C (*n* = 2), A/D (*n* = 1), and C/D (*n* = 10), involving only dogs in pet shops and the veterinary clinic, but not household dogs. Dogs in pet shops and the veterinary clinic harbored assemblages A to D, whereas household dogs examined in this study had only assemblage A.

**Fig. 1:**
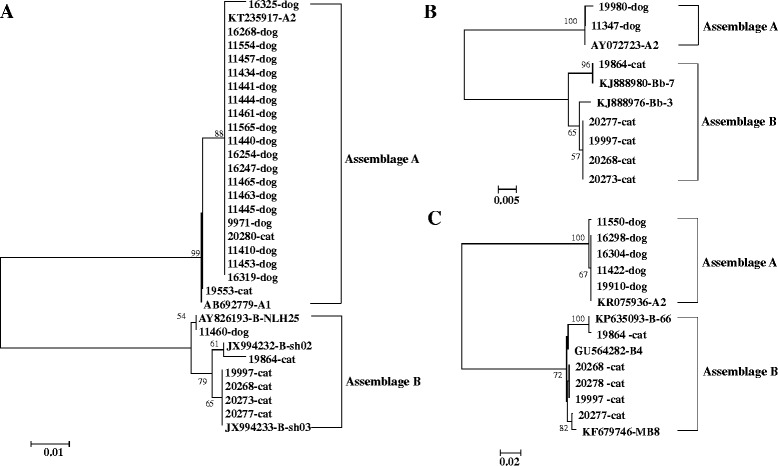
Phylogenetic trees of *Giardia duodenalis* assemblages A and B from dogs and cats as inferred by neighbor-joining analyses of three genetic loci using the Kimura 2-parameter model. Bootstrap values greater than 50 % from 1,000 replicates are shown. **a** Tree based on the glutamate dehydrogenase (*gdh*) gene. **b** Tree based on the β-giardin (*bg*) gene. **c** Tree based on the triose phosphate isomerase (*tpi*) gene

The number of positive specimens belonging to assemblages A and B was 27, 7, and 10 at the *gdh*, *bg*, and *tpi* loci, respectively. Multi-locus sequencing analysis further identified several subtypes of assemblages A and B. At the *gdh* locus (Fig. 1a), sequence alignment of the 21 assemblage A sequences obtained from this study and reference sequences showed that 18 canine specimens and one feline specimen belonged to subtype A2 (KT235917), one canine specimen 16325 was similar to subtype A2 (KT235917) with 2 SNPs (G to A substitution at position 95 and G to A substitution at position 283), and one feline specimen 19553 belonged to subtype A1 (AB692779). In addition, six specimens belonged to assemblage B, including one canine specimen belonging to subtype B-NLH25 (AY826193), four feline specimens belonging to subtype B-sh03 (JX994233), and one feline specimen similar to subtype B-sh02 (JX994232) with 2 SNPs (C to T substitution at position 72 and G to A substitution at position 75). At the *bg* locus (Fig. 1b), two canine specimens belonged to subtype A2 (AY072723) with one sequence of 19980 having 1 SNP (A to T substitution at position 149) from AY072723. One feline specimen belonged to subtype Bb-7 (KJ888980), and the other four feline specimens were similar to subtype Bb-3 (KJ888976) with 2 SNPs (G to A substitution at position 93 and T to C substitution at position 186). At the *tpi* locus (Fig. 1c), four canine specimens belonged to subtype A2 (KR075936) and one canine specimen 11550 was similar to A2 with 2 SNPs (T to C substitution at position 213 and A to G substitution at position 416). In addition, five feline specimens belonged to assemblage B, including three specimens similar to subtype B4 (GU564282) with 2 SNPs (C to T substitution at position 257 and T to C substitution at position 424), one specimen similar to subtype B-66 (KP635093) with 1 SNP (G to A substitution at position 491), and one specimen similar to subtype MB8 (KF679746) with 2 SNPs (G to A substitution at position 266 and G to A substitution at position 283).

**Fig. 2:**
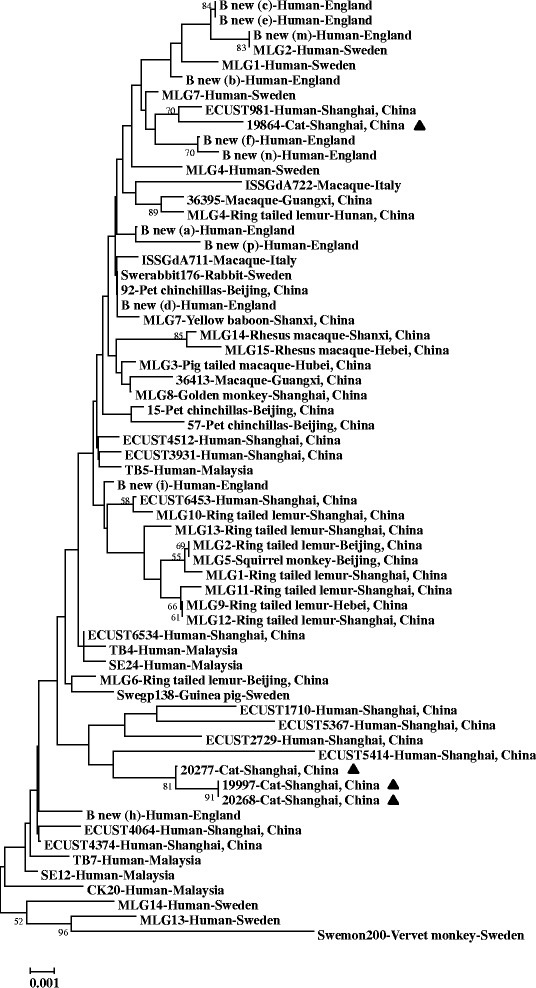
Phylogenetic relationship among multi-locus sequence subtypes of *Giardia duodenalis* assemblage B from this and previous studies as inferred by a neighbor-joining analysis of concatenated sequences of the *gdh*, *bg*, and *tpi* loci, based on genetic distances calculated by Kimura 2-parameter model. Bootstrap values greater than 50 % from 1,000 replicates are shown. The four specimens from cats in this study are indicated by filled triangles

Additional multi-locus typing was conducted based on the concatenated sequences of the *gdh*, *bg*, and *tpi* loci. For assemblage A-positive specimens, multi-locus typing was impossible because none of the specimens were successfully amplified and sequenced at all three loci. For assemblage B-positive specimens, four of seven specimens were successfully sequenced at all three loci and all of them were from cats. An alignment of the concatenated sequences was made together with reference sequences from previous reports of assemblage B in humans, non-human primates, rabbits and guinea pigs from England, Sweden, Italy, Malaysia, and China [16, 21, 23–30]. In a neighbor-joining analysis, three specimens (19997, 20268, and 20277) from cats in Shanghai grouped with four specimens from humans in Shanghai, and another feline specimen (19864) was placed into another cluster containing human specimens from England, Sweden, and Shanghai, China (Fig. 2).

### Mixed infections of *Cryptosporidium* spp., *E. bieneusi* and *G. duodenalis* in dogs and cats

Mixed infections of these three pathogens in dogs and cats are shown in Table 3. Co-infection of the three pathogens was found in one dog. Thirteen dogs and one cat were infected concurrently by *C. canis* (dogs) or *C. felis* (the cat) and *G. duodenalis*, whereas one cat was infected by *C. felis* and *E. bieneusi*. The numbers of co-infections of *E. bieneusi* and *G. duodenalis* were 10 and 3 in dogs and cats, respectively. Dogs in the veterinary clinic (11.5 %) had a significantly higher co-infection rate than dogs in pet shops (4.7 %; *P <*0.05) and household dogs (2.0 %; *P <*0.05). Cats in the animal shelter (5.0 %) had a slightly higher occurrence of co-infection than cats in pet shops (2.5 %; *P* > 0.05).

**Table 3:** Mono- and co-infections of *Cryptosporidium* spp., *Enterocytozoon bieneusi* and *Giardia duodenalis* in dogs and cats in Shanghai, China

Infection types	No. of animals		Pathogens		
	Dogs	Cats	*Cryptosporidium* spp.	*E. bieneusi*	*G. duodenalis*
Single infection	25	4	+	−	−
	18	5	−	+	−
	103	17	−	−	+
	315	129	−	−	−
Co-infection	1	0	+	+	+
	0	1	+	+	−
	13	1	+	−	+
	10	3	−	+	+

## Discussion

Pets such as dogs and cats are intimate companions of humans, but might be reservoirs of human pathogens. To assess the zoonotic potential of *Cryptosporidium* spp., *E. bieneusi* and *G. duodenalis* from pets, we conducted a molecular epidemiological survey of these pathogens in dogs and cats in Shanghai, China, where comparable data from humans are available. We also assessed the extent of cross-transmission of these pathogens between dogs and cats, which often share the same habitat.

Various infection rates of *Cryptosporidium* spp., *E. bieneusi* and *G. duodenalis* were observed in dogs and cats living in different hygiene conditions. When the age groups of < 6 months and 6–24 months were compared, there was no significant age-associated difference in infection rates of the three pathogens in dogs and cats. This is in concordance with observations in some earlier studies in Japan, Colombia, Colorado and one survey in Henan, China [14, 31–33]. In contrast, a few studies in Henan, Shenzhen, Guangzhou and Qinhuangdao, China have shown noticeable age-associated differences in the occurrence of these pathogens in dogs or cats [10, 13, 14]. By living conditions, infection rates of the three pathogens in dogs in pet shops and one clinic were significantly higher than the one in household dogs, suggesting that overcrowding and insufficient sanitary control in veterinary clinics and pet shops may be risk factors in the transmission of these pathogens in pet dog. In addition, a significantly higher occurrence of co-infections with these pathogens was also found in dogs in pet shops and the clinic than in household dogs, supporting the conclusion on increased transmission of pathogens in places where overcrowding occurs. Similarly, a higher occurrence of these pathogens was also observed in cats in one animal shelter than in pet shops. Thus, efforts should be made in veterinary clinics and pet shops to improve sanitary control.

As in most previous studies elsewhere [7], we have identified the occurrence of only *C. canis* in dogs and *C. felis* in cats. In one recent study in Heilongjiang Province in China, several *Cryptosporidium* spp. were detected in dogs and cats, including *C. canis*, *C. felis*, *C. parvum* and *C. ubiquitum* [8]. *Cryptosporidium canis* and *C. felis* have been frequently found in humans in developing countries and are among the five most common human-pathogenic species of  *Cryptosporidium* [3]. In China, *C. canis* and *C. felis* were detected in some human cases in Shanghai [15], indicating that zoonotic transmission of these two species is possible. However, all *C. canis* infections were identified in this study in pet shops and the veterinary clinic, and none were identified in 102 household dogs examined, suggesting the zoonotic potential of the parasite is minimal in urban households.

Similar to *Cryptosporidium* spp., a low genetic heterogeneity of *E. bieneusi* was also observed in dogs and cats in the present study. Unlike the finding of numerous *E. bieneusi* genotypes in similar studies in other parts of China, including Heilongjiang, Sichuan, Chongqing, Shaanxi, Jilin, and Henan Provinces [8, 14, 34], we have identified only genotypes PtEb IX and D in dogs and genotypes Type IV and D in cats. The dominant occurrence of genotype PtEb IX (28/29) in canine specimens has been reported in dogs worldwide and the genotype is considered the most common dog-adapted *E. bieneusi* genotype [1]. The two genotypes (Type IV and D) in cats belong to Group 1 and are the most common zoonotic genotypes, having been found in humans, nonhuman primates, dogs, cats, and some livestock in China [8, 35–37]. Our previous studies in Shanghai identified genotype D in an in-hospital child and genotypes D and Type IV in wastewater [16, 17]. Elsewhere in China, these two genotypes are common in HIV/AIDS patients in Henan Province [37].

*Giardia duodenalis* assemblages C and D in dogs and assemblage F in cats have been identified as the most common genotypes in pet animals around the world [2]. In this study, in addition to C and D, assemblage A was also found in many dogs, in agreement with two previous surveys in Guangdong Province [10, 11]. Because of the zoonotic potential of assemblage A, dog-to-human transmission of giardiasis might be possible in China. Similarly, in addition to assemblage F, the zoonotic assemblage B was commonly found in cats in this study, which was only occasionally detected in cats in Australia and Europe but has never been reported in cats in China [2, 8, 9].

Multi-locus sequence typing was used in genetic characterization of *G. duodenalis* in this study. The *gdh*, *bg*, and *tpi* loci showed various PCR amplification rates and somewhat inconsistent typing results, which is similar to most previous studies on multi-locus typing of *G. duodenalis* [21, 38]. At each locus, almost all assemblage A-positive specimens from dogs were identified as subtype A2 (Fig. 1), which was reported for the first time in pet dogs in China. Earlier studies demonstrated that the majority of assemblage A infections in dogs worldwide belonged to sub-assemblage AI [2]. In our previous studies in Shanghai, the subtype A2 was also found to be dominant in human cases and waste-water [16, 17]. In addition, phylogenetic analysis of concatenated sequences from the three genetic loci suggested that assemblage B subtypes in cats in Shanghai were closely related to those in humans in the same city [16].

Despite the occurrence of some human-pathogenic *Cryptosporidium* species and *E. bieneusi* and *G. duodenalis* genotypes in dogs and cats, the distribution of these pathogens is generally different between dogs and cats examined in this study. Dogs are mainly infected with *C. canis*, *E. bieneusi* genotype PtEb IX and *G. duodenalis* assemblages A, C and D. In contrast, cats in the same area or even the same pet shops are mostly infected with *C. felis*, *E. bieneusi* genotypes Type IV and D and *G. duodenalis* assemblages B and F. This suggests that there is minimum inter-species transmission of *Cryptosporidium* spp., *E. bieneusi* and *G. duodenalis* between dogs and cats.

## Conclusions

This study revealed that living condition is a factor affecting the transmission of *Cryptosporidium* spp., *E. bieneusi* and *G. duodenalis* in dogs and cats in Shanghai, China. The presence of shared species or genotypes of *Cryptosporidium* spp., *E. bieneusi* and *G. duodenalis* in pet animals and humans in the same geographic area suggests that pet animals could be potential reservoirs for human infections of these pathogens. Nevertheless, data obtained from the study also suggest that inter-species transmission of these pathogens is minimal between dogs and cats living in the same area or habitat. Further studies involving extensive sampling of humans and animals living in the same households and better epidemiological designs are needed to elucidate the role of companion animals in the epidemiology of cryptosporidiosis, microsporidiosis and giardiasis.

### Ethics statement

Fecal specimens in the study were collected under the permission of pet owners, the veterinary clinic owner, and pet shop owners. The research protocol was reviewed and approved by the Ethics Committee of East China University of Science and Technology.

## Abbreviations

*bg*: β-giardin
*gdh*: glutamate dehydrogenase
ITS: internal transcribed spacer
SNP: single nucleotide polymorphism
SSU: small subunit
*tpi*: triosephosphate isomerase
